# The Effect of Polyvinyl Alcohol Addition on the Optical Properties and Oxygen Detection Performance of Titanium Dioxide and Methylene Blue Nanocomposite Colorimetric Indicators

**DOI:** 10.3390/polym16101400

**Published:** 2024-05-14

**Authors:** Kanokthip Boonyarattanakalin, Praphaporn Rattan, Maneerat Songpanit, Sutee Chutipaijit, Hideyuki Okumura, Keiichi N. Ishihara, Wanichaya Mekprasart, Wisanu Pecharapa

**Affiliations:** 1 College of Materials Innovation and Technology, King Mongkut’s Institute of Technology Ladkrabang, Bangkok 10520, Thailand; kanokthip.bo@kmitl.ac.th (K.B.); 65116007@kmitl.ac.th (P.R.); 66116007@kmitl.ac.th (M.S.); sutee.ch@kmitl.ac.th (S.C.); wisanu.pe@kmitl.ac.th (W.P.); 2 Graduate School of Energy Science, Kyoto University, Yoshida Honmachi, Sakyo, Kyoto 606-8501, Japan; okumura@energy.kyoto-u.ac.jp; 3 Open Innovation Institute, Kyoto University, Yoshida Honmachi, Sakyo, Kyoto 606-8501, Japan; ishihara.keiichi.6w@kyoto-u.ac.jp

**Keywords:** colorimetric, milling process, nanocomposite, oxygen indicator, TiO_2_/methylene blue

## Abstract

In this study, we investigated the impact of polyvinyl alcohol (PVA) incorporation on the optical properties and oxygen detection performance of a titanium dioxide/methylene blue (TiO_2_/MB) nanocomposite colorimetric indicator for packaging applications. The nanocomposite was synthesized via mechanical milling of TiO_2_ nanoparticles with MB and citric acid. PVA, at varying concentrations (0, 3, 9, and 14 wt%), was introduced during the wet milling process to produce a homogeneous composite film. Spin coating was employed to fabricate TiO_2_/MB nanocomposite films for oxygen detection evaluation. The influence of PVA loading on the films’ chemical functionalities and surface morphologies was assessed using Fourier-transform infrared spectroscopy (FTIR) and field-emission scanning electron microscopy (FE-SEM). The indicator’s activation process, involving a color change between bleached and colored states, and its recovery time were monitored via optical imaging and UV-VIS-NIR spectrophotometry. The results revealed that a PVA content of 9 wt% yielded well-defined films with enhanced stability of the TiO_2_/MB nanocomposite’s oxygen detection performance.

## 1. Introduction

Traditional packaging serves multiple purposes beyond marketing, encompassing product protection from external factors, providing containers of various sizes and shapes with ease of use, and ensuring long shelf life for consumer convenience. The increase in urban populations and changing lifestyles have driven the demand for ready-to-use, high-quality, and safe packaging in food distribution systems [1]. This innovation necessitates addressing various factors such as temperature, oxygen, carbon dioxide, and toxins for effective food containment and preservation. Oxygen plays a crucial role in food spoilage. Food spoilage microorganisms thrive under aerobic conditions [2]. Consequently, food packaging techniques such as vacuum and modified atmosphere packaging (MAP) are employed with the aim of removing air or replacing it with specific gases to extend shelf life [3,4]. However, these methods have limitations. Leaks can occur during handling, transport, and preparation, compromising their effectiveness. Intelligent packaging with oxygen indicators offers a solution by monitoring oxygen levels inside the package. These indicators provide consumers with valuable information about package integrity and potential leaks [5]. There are three main types of oxygen indicators based on their specific detection mechanisms. First, electrochemical indicators: these indicators rely on electrochemical principles, requiring two electrodes, an electrolyte solution, and a separating membrane. While they offer insights into oxygen gas conditions, food quality, and packaging integrity, they are expensive, require specific user expertise, and can damage the packaging itself [6,7]. Second, optical indicators: these indicators utilize luminescent properties from materials such as long-delay fluorescent or phosphorescent dyes encapsulated in a solid polymer matrix. They enable oxygen monitoring without causing harm to the sample. However, the luminance materials often include toxic elements such as ruthenium, palladium(II), and platinum(II)–porphyrin, rendering them unsuitable for food packaging applications [8]. Third, colorimetric indicators: these indicators consist of a redox dye, a sacrificial electron donor, and a photocatalyst semiconductor. Oxygen’s presence on the indicator’s surface triggers a color change through an oxidation reaction. Colorimetric indicators offer advantages such as low cost, visual assessment by the naked eye, and the use of non-toxic materials, making them ideal for food packaging [9,10].

Metal oxides, particularly titanium dioxide (TiO_2_), are attractive photocatalyst semiconductors in colorimetric indicators due to their biocompatibility and strong UV light absorption properties [11]. However, TiO_2_ powder is insoluble, leading to non-homogeneous films. This issue can be addressed by incorporating polymers or binders to modify the film surface, promoting smoothness and homogeneity. Compared to metallic or ceramic binder types, polymeric binders offer several advantages. Metallic binders are often expensive and may not be compatible with the chemical functionalities needed for optimal oxygen detection. Inorganic ceramic binders, such as colloidal silica, typically require a curing temperature exceeding 800 °C to activate their binder properties [12]. Moreover, binders from hybrid nanostructures can be complex to synthesize and may introduce unwanted properties that affect the indicator’s performance. Thus, polymeric binders offer as much versatility in film fabrication as those utilized for oxygen indicators. The mechanism of polymeric binders can be proposed in various environments as conventional binder drying through a physical process, binder crosslinking through cation–anion interaction, crosslinking with hardener molecules, or a reactive polymeric or oligomeric material prone to crosslinking under UV activation [13]. Polyvinyl alcohol (PVA) has emerged as a promising polymeric binding agent for oxygen indicators due to its unique properties, including good adhesion, corrosion resistance, a lack of odor, a non-toxic nature, and environmental friendliness [14]. Research by Hye-Kyoung Park et al. suggests that PVA’s numerous hydroxyl groups make it an excellent binder, enhancing adhesion strength through strong hydrogen bonds between active materials and the current collector. This feature significantly improves the electrochemical properties of high-capacity anodes [15]. PVA’s versatility extends beyond its application in this study, with it finding use in various commercial, medical, and food packaging materials [16].

In this study, we investigated the impact of PVA addition on the optical properties and oxygen detection efficiency of a TiO_2_/MB colorimetric oxygen indicator. We explored the potential of a composite film containing TiO_2_ nanoparticles (photocatalyst), methylene blue (redox dye), citric acid (sacrificial electron donor), and PVA (binder polymer). The effect of the addition of PVA was monitored through surface morphology and chemical bonding analyses. Techniques such as optical imaging and spectroscopy were employed to characterize oxygen detection by the film composite with varying PVA loadings. The oxygen indicator performance of the TiO_2_/MB nanocomposite was tested in a vacuum system to confirm oxygen-induced color switching. Lastly, we assessed the oxygen detection properties of the TiO_2_/MB indicator in different container volumes to assess its suitability in packaging applications.

## 2. Materials and Methods

### 2.1. Materials

Commercially available TiO_2_ nanopowder, used as a metal oxide photocatalyst semiconductor, was purchased from P25 Titanium dioxide nanopowder, Nanografi company (Jena, Germany). Methylene blue with analytical-grade reagent as a redox dye was purchased from LOBA Chemie (Mumbai, India). Citric acid (analytical reagent grade) and polyvinyl alcohol (technical grade) with a molecular weight of 77,000 to 82,000 g/mol were supplied by the Ajax Finechem company (New South Wales, Australia). All chemicals were used as received without further purification.

### 2.2. Synthesis of TiO_2_/MB Nanocomposite Suspension

The TiO_2_/MB nanocomposite suspension was synthesized using a high-energy mechanical milling process, following the procedure described in our previous work [17]. First, 3 g of TiO_2_ P25 powder, 12.5 mg of MB, and 0.15 g of citric acid were added to milling containers at a mass ratio of composite precursors to zirconia grinding media ball of 1:10. The dry milling process was set to a speed of 250 rpm for 30 min. PVA solutions were then prepared by dissolving PVA powder at concentrations of 0, 1, 3, and 5% (*w*/*v*) in deionized water. Afterward, 10 mL of each PVA solution (with a specified concentration) was added to the TiO_2_/MB composite powder, and the mixture was additionally milled during the wet process for 45 min at the same speed as the dry process. Finally, the homogeneous blue ink suspension of the TiO_2_/MB nanocomposite contained PVA concentrations of 0, 3, 9, and 14 wt%.

### 2.3. Preparation of TiO_2_/MB Nanocomposite Films

The TiO_2_/MB nanocomposite suspension with different PVA concentrations under all conditions was coated on a glass slide (with an area of 2.5 × 2.5 cm^2^) using the spin coating technique with a spin speed of 3000 rpm and 5 film layers. Afterward, the TiO_2_/MB composite films were dried in ambient air at 100 °C for 30 min and exposed to UVA irradiation (λ_max_ at 360 nm) to activate them in a photobleached state. Lastly, the bleached nanocomposite films returned to a blue color after oxygen detection within an air environment at room temperature.

### 2.4. Characterization

The surface morphology, cross-section, and roughness of the TiO_2_/MB nanocomposite films with varying PVA content were monitored using a field emission scanning electron microscope (FE-SEM; JSM-7001F, JOEL, Tokyo, Japan) with a magnification of ×10,000 and an atomic-force microscope (AFM; Hitachi 5300E, Hitachi High-Tech Corporation, Tokyo, Japan). Chemical functional group and optical properties in the composite films were analyzed using an ATR-FTIR spectrometer (Spectrum Two FT-IR Spectrometer, PerkinElmer Scientific, Shelton, CT, USA) and a UV-VIS-NIR spectrophotometer (UH4150, Hitachi High-Tech Corporation, Tokyo, Japan) in reflectance mode. To determine the color-changing state, photobleached activity (initial state), and recovery time under oxygen detection (color state) of the activated TiO_2_/MB nanocomposite indicator were monitored using a timer and surveillance camera. The phase identification of the TiO_2_/MB nanocomposite films before/after UV activation and oxygen detection was examined using the X-ray diffraction technique (XRD; TTRAX III, Rigaku corporation, Tokyo, Japan) in the 2θ range of 15°–70°. The oxygen detection performance of the TiO_2_/MB indicators was operated under a pressure of 150 mbar to detect its color-switching on the indicator surface at room temperature. The TiO_2_/MB indicator, with 9 wt% PVA loading, was monitored to assess oxygen detection within the different container volumes for the possibility of packaging application.

## 3. Results and Discussion

### 3.1. Structural and Chemical Properties of TiO_2_/MB Nanocomposite Films

**Figure 1:**
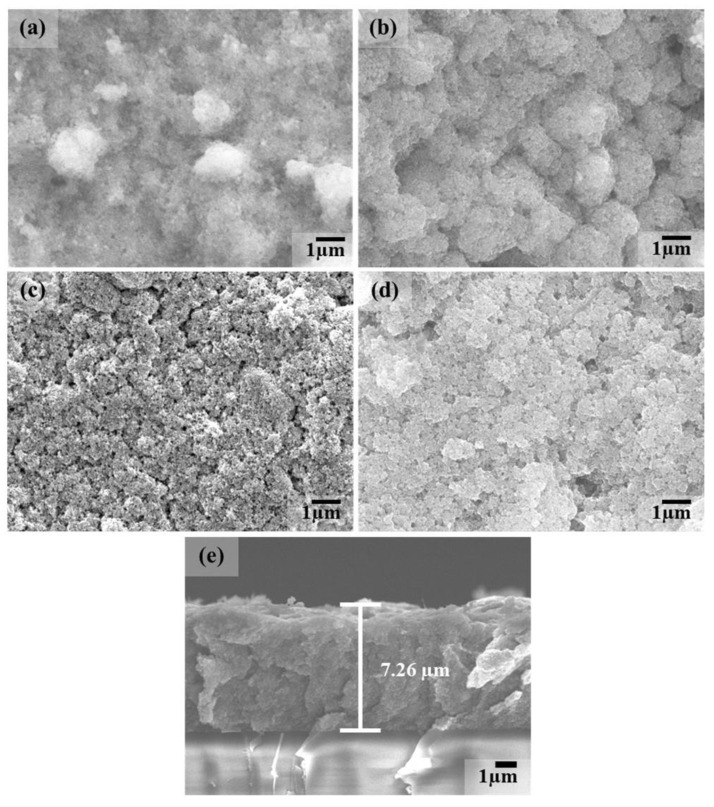
Surface morphologies of TiO_2_/MB nanocomposite films prepared using various PVA content for the (**a**) as-prepared film and at (**b**) 3 wt%, (**c**) 9 wt%, and (**d**) 14 wt%, and (**e**) a cross-sectional image of the TiO_2_/MB nanocomposite film at 9 wt% PVA.

**Figure 2:**
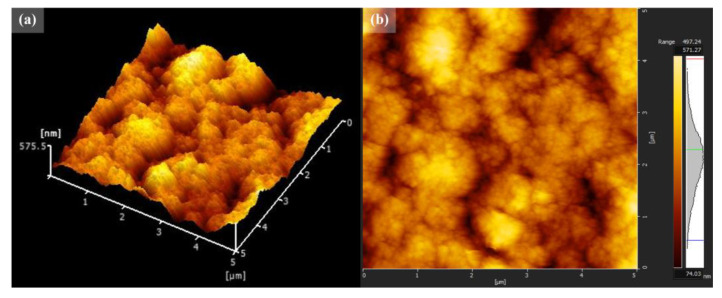
(**a**) The 3D-AFM images and (**b**) surface morphology of the TiO_2_/MB nanocomposite film at 9 wt% PVA.

**Figure 3:**
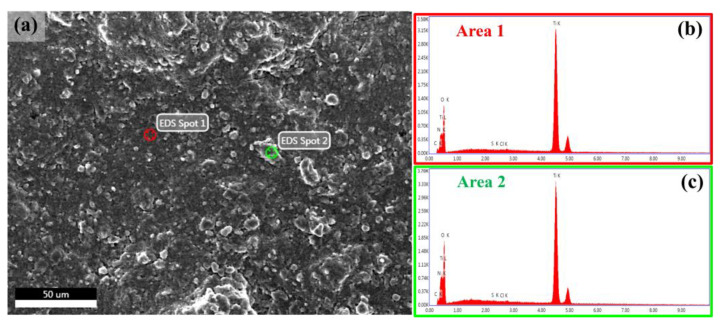
EDS analysis of the TiO_2_/MB nanocomposite film at 9 wt% PVA, with analysis performed in two distinct areas with (**a**) the scanned area and the analyzed spectrum of (**b**) the first area and (**c**) the second area.

FE-SEM images of TiO_2_/MB nanocomposite films with varied PVA content are shown in Figure 1. The morphology of the TiO_2_/MB composite films was dense and fully packed due to the increased PVA loading. It can be observed that the film without the PVA polymer, as shown in Figure 1a, shows high particle aggregation and a non-uniform surface. With the loading PVA concentration at 3 wt%, as shown in Figure 1b, the uniformity of the film surface on the TiO_2_/MB composite film increased and a certain degree of roughness was still present on the film. For the addition of PVA content at 9 wt%, the uniformity on the nanocomposite film surfaces significantly increased and less particle aggregation was observed, as shown in Figure 1c. Moreover, the structure on the film surface with loading PVA at 14 wt%, as seen in Figure 1d, was identical to the film condition with loading PVA at 9 wt% in the composite. However, some agglomerated areas of the composite powder observed on the film surface resulted from high PVA binder concentrations. The cross-section of the 9 wt% PVA in the TiO_2_/MB composite film is illustrated in Figure 1e. The film thickness of the TiO_2_/MB composite film after five-layer deposition was measured as approximately 7.26 µm. Our previous work shows that film thickness is influenced by rotation speed and film layer deposition [18]. Consistent layer deposition achieved similar thicknesses with other PVA concentrations. Hence, a homogenous composite with good particle dispersion, a smooth film surface, and uniform film formation was obtained and improved upon through the optimized ratio of PVA addition to the TiO_2_/MB nanocomposite. The surface roughness of the TiO_2_/MB composite film at 9 wt% PVA was roughly estimated to be 86 nm, as indicated in Figure 2. However, the film morphology using high-resolution 3D AFM imaging revealed significant surface roughness attributed to the spin-coating process by the powder suspension. Additionally, EDS analysis of the TiO_2_/MB nanocomposite film with 9 wt% PVA was conducted in different areas, as depicted in Figure 3. The elemental spectra obtained from the composite film reported the compatible amounts of carbon (C), nitrogen (N), oxygen (O), chlorine (Cl), and titanium (Ti) atoms across these areas. However, a slightly increased signal intensity was observed in the second area due to localized clustering caused by the composite aggregation on the film surface. This result suggests that the uniform distribution of the TiO_2_/MB composite could be achieved via the milling process and the effective dispersion of the PVA binder.

**Figure 4:**
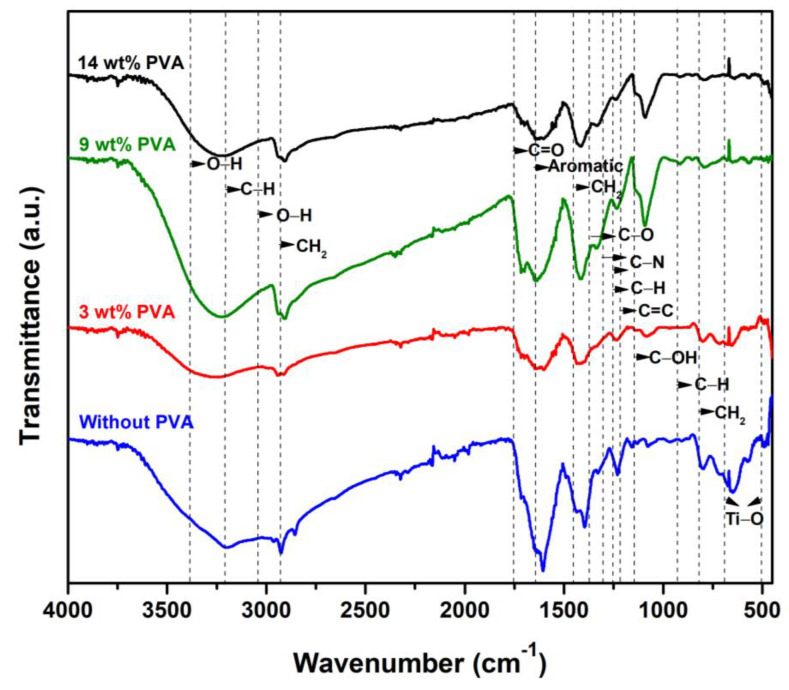
FT-IR spectra of the TiO_2_/MB nanocomposite with varied PVA content.

The functional groups of the TiO_2_/MB nanocomposite film were characterized via FT-IR spectroscopy techniques, as depicted in Figure 4. The FT-IR spectrum of TiO_2_/MB composite films based on varying PVA concentrations showed similar patterns to the as-prepared film (with no PVA binder). The significant peaks of titanium dioxide located at 483 cm^−1^ and 691 cm^−1^ were related to the Ti-O stretching bond. In the methylene blue structure, the significant peaks observed at 885 cm^−1^, 1220 cm^−1^, and 1249 cm^−1^ were associated with the vibration of C-H and C-N bonds. The peak positions at 1179 cm^−1^, 1605 cm^−1^, 3030 cm^−1^, and 3370 cm^−1^ were possibly related to the C=C bond vibration from the aromatic ring and the vibration of C-H and O-H bonds. The chemical bonding of citric acid was found at 779 cm^−1^, 1106 cm^−1^, 1330 cm^−1^, 1722 cm^−1^, and 3290 cm^−1^ and was associated with CH_2_ rocking, C-O stretching, C-OH stretching, C=O stretching, and O-H stretching, respectively [19]. The different peaks of the TiO_2_/MB nanocomposite films with PVA loading showed varying peaks at 1425 cm^−1^, 1690 cm^−1^, and 2917 cm^−1^ belonging to the intense peaks of the PVA polymer. Significant peaks of PVA were found in the composite films related to C-H bending from CH_2_, C=O carbonyl stretching, and CH_2_ asymmetric stretching vibration. However, the broad peak in the range of the wavenumber of 3200 to 3400 cm^−1^ in each pattern corresponded to the vibration of the O-H stretching bonds from the hydroxyl groups on the sample surface [20].

### 3.2. Colorimetric Indicator Efficiency of TiO_2_/MB Nanocomposite Films

Photographs of TiO_2_/MB nanocomposite films before and after UVA activation, along with bleaching time observations on the film surfaces with different PVA concentrations, are shown in Table 1. In the case of pre-UVA irradiation, the TiO_2_/MB nanocomposite film with the absence of PVA binder exhibited an indigo-blue shading. In addition, high roughness on the film surface originated from particle aggregation due to the absence of a polymer binder in the film’s fabrication. Following UV irradiation, the composite film changed to a colorless state in 34 s in the clear area. The remaining blue areas on the film surface were observed via the presence of thick layers from the particle agglomeration on the substrate. To resolve this issue, the addition of the PVA polymer to the composite was chosen as a binder to enhance the homogeneous surface film and reduce particle aggregation in the film-casting method. The light-blue shade on the films was obtained via increased PVA loading in the composite mixture. The color change in the TiO_2_/MB composite films by PVA contents of 3, 9, and 14 wt% occurred at 30, 28, and 100 s. Through analysis of the FE-SEM images, good particle dispersion, a homogenous film, and a flat surface developed due to the increase in PVA concentration which led to a thin layer and facilitated UV activation on its surface. Moreover, the bleached time of the film with PVA concentrations of 3 and 9 wt% was short compared with the absence of PVA polymer in the film because of good particle dispersion and the homogenous film surface. In addition, the extended bleached time of the composite film with a PVA concentration of 14 wt% under UV irradiation was obtained due to the cover of excessive PVA concentration on titanium dioxide and methylene blue particles. This result was related to the retardant of the interaction between titanium dioxide and methylene blue under UV activation [21].

**Figure 5:**
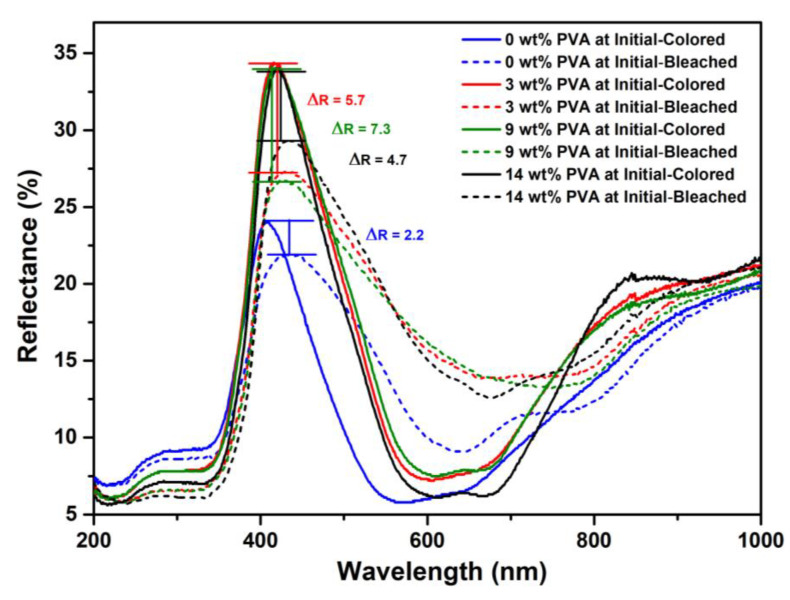
DRS spectra of TiO_2_/MB nanocomposite films with different PVA content before and after UV irradiation ((—) before and (---) after UV irradiation).

The optical analysis of TiO_2_/MB nanocomposite films in color/bleached state via DRS spectra is demonstrated in Figure 5. The performance of the TiO_2_/MB oxygen indicator was evaluated based on the colored/bleached state mechanism of oxygen detection. The high reflectance in the range of 350 to 600 nm, as shown in all spectra, corresponded to the presence of methylene blue in the TiO_2_/MB composite films in the initial or colored state (shown with straight lines). Moreover, the dotted lines indicate the state of the bleached or colorless area on the film surface. Thus, the difference in reflectance spectra (ΔR) between the initial and bleached state of the TiO_2_/MB oxygen indicator at λ_max_ of methylene blue absorption was calculated to verify its color-switching mechanism. The lowest reflectance difference in the as-prepared TiO_2_/MB nanocomposite film showed a value of 2.2. In contrast, the other conditions of the TiO_2_/MB composite films with different PVA concentrations at 3, 9, and 14 wt% exhibited the ΔR values of 5.7, 7.3, and 4.7 to estimate the color-switching on the indicators. Thus, the PVA concentration of 9 wt% was determined to be suitable for TiO_2_/MB film fabrication owing to it demonstrating the highest ΔR. Moreover, this significant ΔR value was also associated with a noticeable difference in the color of the TiO_2_/MB nanocomposite film before and after UV irradiation.

The oxygen detection performance of TiO_2_/MB oxygen indicators with varying PVA contents in the nanocomposite was determined under ambient air conditions through the use of photographs taken at different time intervals, as detailed in Table 2. The cyan percentage of blue shading under oxygen detection on the indicator surface was investigated to identify its status under oxygen detection. The initial state of all TiO_2_/MB oxygen indicators was in the cyan percentage of almost 100% owing to the methylene blue structure in the composite. Following UVA irradiation, the TiO_2_/MB composite layer changed from a colorless to a bleached state due to the influence of hydrogen bonding change in methylene blue to a leuco-methylene blue (LMB) structure, owing to the photocatalytic and oxidation processes between TiO_2_ and the methylene blue material. Therefore, the colorless indicators with an LMB structure, which were initially labeled as indicating the absence of oxygen gas, changed to a blue shade (colored state) after interaction with oxygen gas in ambient air. This color switching on the indicator occurred due to the oxidation of the LMB structure, with the interaction of oxygen molecules leading to the complete formation of the MB structure [22]. Meanwhile, the oxygen detection performance of the modified indicators with varying PVA contents exhibited the same pattern, with a complete transition to a blue color observed at around 12 h. Thus, the amount of PVA loading in the TiO_2_/MB nanocomposites did not affect the oxygen detection performance. The fastest color recovery time of blue shading on the oxygen indicator was observed on the bare TiO_2_/MB indicator without the PVA binder, as oxygen gas was able to directly interact with the indicator under ambient air conditions. Complete decoloration to a dark blue on the bare composite film was achieved within 5 min. Moreover, the retardation of the blue color on the indicator assisted by varying PVA content was noted. Complete blue shading was attained after 24 h of oxygen detection under ambient air conditions. Therefore, the PVA binder exerted an influence on color switching on the indicator surface.

**Figure 6:**
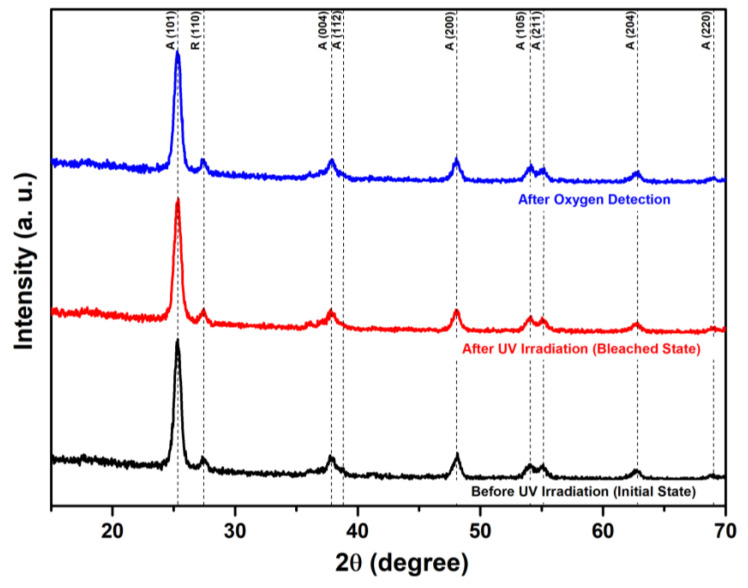
XRD patterns of the 9 wt% PVA composite films before/after UV irradiation and after oxygen detection.

The XRD patterns of 9 wt% PVA in the TiO_2_/MB nanocomposite films before/after UV irradiation and exposure to an oxygen detection environment are presented in Figure 6. The overall patterns were identical to the main phase of TiO_2_ crystallinity. The observed peaks in all patterns at 2θ of 25.3°, 37.8°, 38.7°, 48.1°, 53.9°, 55.1°, 62.7°, and 68.7° corresponded to the anatase TiO_2_ crystalline planes of (101), (004), (112), (200), (105), (211), (204), and (220) (CSD No. 9008216). Additionally, the peak at 27.5° was associated with the rutile phase of the TiO_2_ structure (CDS No. 80842). These results suggest that the stable crystallinity of the TiO_2_ phase in the composite remained following UV activation and oxygen exposure. Thus, the mechanism of color switching in the TiO_2_/MB composite film is likely attributed to a chemical bonding change from methylene blue to a leuco–methylene blue structure.

**Figure 7:**
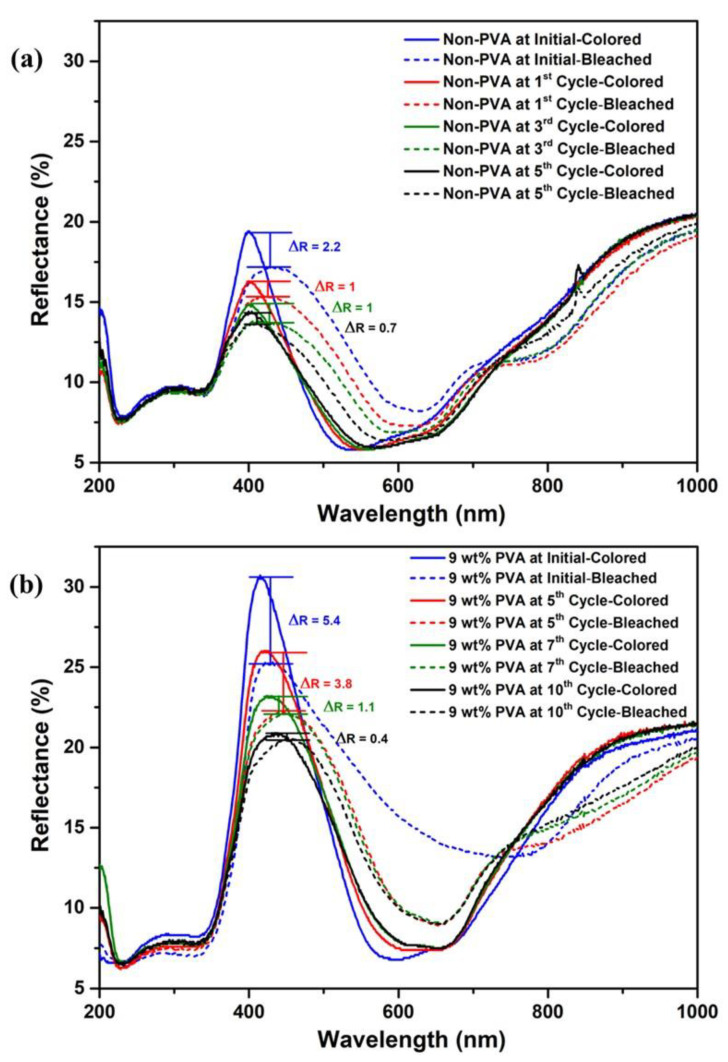
DRS spectra of the reused cycles in TiO_2_/MB nanocomposite films with (**a**) as-prepared film and (**b**) PVA content at 9 wt% before and after UV irradiation ((—) before and (---) after UV irradiation).

The reusability of TiO_2_/MB nanocomposite films, as observed from diffuse reflectance spectra, is illustrated in Figure 7. Initially, the DRS spectra of the as-prepared TiO_2_/MB nanocomposite film in Figure 7a showed a ΔR value of 2.2, as detailed in Figure 4. However, decreased reflectance intensity was observed under repeated UV activation during the first and third cycles. This decrease may be attributed to the photodegradation of redox dye under UV illumination and the capture of oxygen molecules on the film surface to reactivate in a colored state again. The ΔR values of the TiO_2_/MB film in the first and third cycles were identical to one another. After the fifth cycle, the ΔR decreased to 0.7, indicating diminished color-switching in the oxygen indicator performance. For the TiO_2_/MB nanocomposite film with the addition of PVA 9 wt%, as shown in Figure 7b, the initial ΔR value was 5.4 after the color-switching mechanism. Following repeated UV irradiation during the fourth cycle, the reflectance intensity at λ_max_ of methylene blue absorption also decreased, resembling the behavior observed in the sample without a PVA binder. The ΔR values after the fourth, seventh, and tenth cycles decreased to 2.4, 1.1, and 0.4.

**Figure 8:**
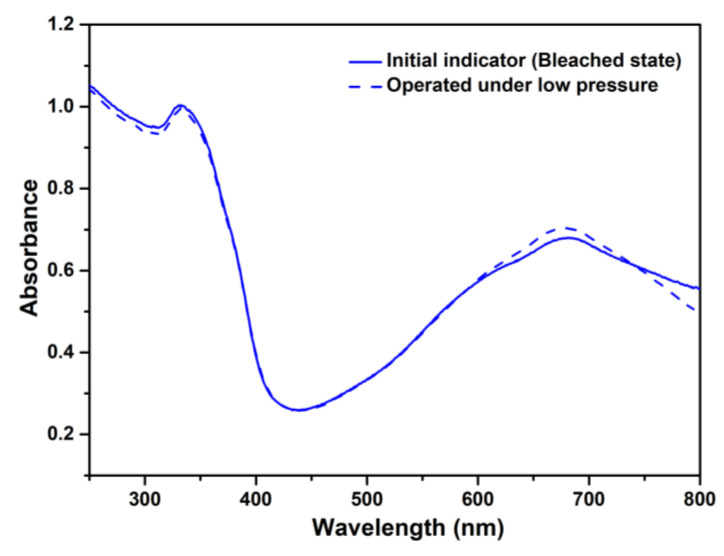
DRS spectra of TiO_2_/MB nanocomposite films with a PVA content at 9 wt% with the initial state and operating under low pressure.

Therefore, the reusable as-prepared TiO_2_/MB nanocomposite film with the absence of PVA polymer was controlled within three cycles. In contrast, the stable TiO_2_/MB composite film with a 9 wt% PVA content reached maximum usability over seven cycles. Thus, optimizing the addition of 9 wt% PVA in the TiO_2_/MB nanocomposite film can improve both its reusability and stability. Figure 8 shows the DRS spectra of TiO_2_/MB nanocomposite films in different environments, including the initial state and operation under a pressure of 150 mbar (low vacuum). Identical spectra of the composite film before/after the use of the low vacuum system were obtained. However, there was an observed increase in absorption intensity, with a peak at 662 nm belonging to methylene blue absorption. The presence of some oxygen molecules from the ambient air in the low vacuum system likely led to a reaction with the leuco-methylene blue molecules, causing it to revert to a colored state. This result confirms that the TiO_2_/MB oxygen indicator requires activation specifically in an oxygen atmosphere.

The performance of the TiO_2_/MB oxygen indicator with the condition of 9 wt% PVA in plastic food containers of different volumes and sizes is presented in Table 3. The results in this section confirm that the color changes to blue in the oxygen gas indicator within the containers depending on the specified air volumes (250 mL, 590 mL, 1200 mL, and 2680 mL) occurring simultaneously. The indicator begins its color change from a bleached state to light blue after 30 min of oxygen detection, gradually intensifying to a darker blue shade over 12 h. Moreover, the cyan percentage on the surface of the TiO_2_/MB oxygen indicators was also monitored. Before UVA irradiation, all oxygen indicators tested in each container showed a cyan percentage of 80–82%. Following UVA activation, the percentage of blue color decreased to 46–48%, indicating the bleached or initial state. During the test under oxygen environments in the containers, the cyan percentage increased to 55% after 30 min and continued to increase. By the end of the experiment, the increase in cyan percentage was identical to the value of the indicator before UVA irradiation at 80–82%, after approximately 10 h for all conditions. Therefore, it was confirmed that the response of the TiO_2_/MB oxygen indicator would be identical in performance regardless of the different amounts of oxygen molecules within the air volumes.

## 4. Conclusions

In this study, we successfully produced a colorimetric oxygen indicator using a TiO_2_/MB nanocomposite with a PVA binder. Optimizing the PVA content to 9 wt% in the film composite yielded a homogenous surface film with excellent performance in oxygen detection. FTIR analysis confirmed that the inclusion of PVA did not alter the core functionalities of the indicator. The optical properties observed via UV-VIS-NIR spectroscopy provided indirect evidence of oxygen detection, as indicated by the color change in methylene blue from its oxidized form to a leuco–methylene blue structure. The PVA binder enhanced repeatability, and the indicator’s response to oxygen remained consistent across the different container sizes. In future research, we will focus on improving sensitivity, long-term stability, real-world food packaging testing, and colorimetric quantification for objective oxygen level assessment and explore the possibility of multigas detection. By addressing these aspects, the results of this research lay the foundation for a practical and reliable tool to ensure food quality and safety.

## Figures and Tables

**Table 1:** The appearance and bleached time of TiO_2_/MB nanocomposite films with varying PVA content under UV activation.

PVA Content (wt%)	Before UVA Irradiation	After UVA Irradiation	Bleached Time (s)
0	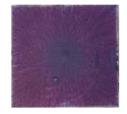	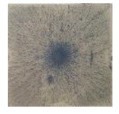	34
3	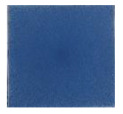	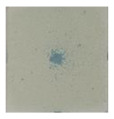	30
9	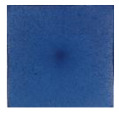	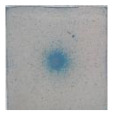	28
14	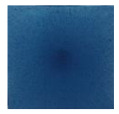	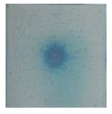	100

**Table 2:** The oxygen detection performance and cyan percentage of TiO_2_/MB nanocomposite films with varying PVA contents under ambient air.

PVA Content (wt%)	Cyan Percentage on the TiO_2_/MB Film Surface								
	Initial State	After UVA Irradiation	Oxygen Detection Times						
			5 min	30 min	2 h	12 h	24 h	36 h	48 h
0	85	46	85	85	85	85	85	85	85
3	99	39	62	66	78	99	100	100	100
9	98	38	55	55	66	94	98	100	100
14	100	47	64	66	79	99	100	100	100

**Table 3:** The appearance and cyan percentage of TiO_2_/MB indicators at 9 wt% PVA in different container volumes.

Container Volume (mL)		Cyan Percentage on the TiO_2_/MB Film Surface							
	Before UVA		Oxygen Detection Times (h)						
		0	0.5	2	4	6	8	10	12
250	82	46	55	67	69	72	76	82	82
590	82	48	57	67	69	72	75	82	82
1200	81	47	55	67	69	72	76	81	81
2680	80	47	53	67	69	72	75	80	80

## Data Availability

Data are contained within the article.
